# Deciphering metabolic heterogeneity in retinoblastoma unravels the role of monocarboxylate transporter 1 in tumor progression

**DOI:** 10.1186/s40364-024-00596-8

**Published:** 2024-05-11

**Authors:** Junjie Tang, Yaoming Liu, Yinghao Wang, Zhihui Zhang, Jiahe Nie, Xinyue Wang, Siming Ai, Jinmiao Li, Yang Gao, Cheng Li, Chao Cheng, Shicai Su, Shuxia Chen, Ping Zhang, Rong Lu

**Affiliations:** State Key Laboratory of Ophthalmology, Zhongshan Ophthalmic Center, Sun Yat-sen University, Guangdong Provincial Key Laboratory of Ophthalmology and Visual Science, Guangzhou, 510060 China

**Keywords:** Retinoblastoma, Cancer metabolism, Monocarboxylate transporter 1, AZD3965

## Abstract

**Background:**

Tumors exhibit metabolic heterogeneity, influencing cancer progression. However, understanding metabolic diversity in retinoblastoma (RB), the primary intraocular malignancy in children, remains limited.

**Methods:**

The metabolic landscape of RB was constructed based on single-cell transcriptomic sequencing from 11 RB and 5 retina samples. Various analyses were conducted, including assessing overall metabolic activity, metabolic heterogeneity, and the correlation between hypoxia and metabolic pathways. Additionally, the expression pattern of the monocarboxylate transporter (MCT) family in different cell clusters was examined. Validation assays of MCT1 expression and function in RB cell lines were performed. The therapeutic potential of targeting MCT1 was evaluated using an orthotopic xenograft model. A cohort of 47 RB patients was analyzed to evaluate the relationship between MCT1 expression and tumor invasion.

**Results:**

Distinct metabolic patterns in RB cells, notably increased glycolysis, were identified. This metabolic heterogeneity correlated closely with hypoxia. MCT1 emerged as the primary monocarboxylate transporter in RB cells. Disrupting MCT1 altered cell viability and energy metabolism. In vivo studies using the MCT1 inhibitor AZD3965 effectively suppressed RB tumor growth. Additionally, a correlation between MCT1 expression and optic nerve invasion in RB samples suggested prognostic implications.

**Conclusions:**

This study enhances our understanding of RB metabolic characteristics at the single-cell level, highlighting the significance of MCT1 in RB pathogenesis. Targeting MCT1 holds promise as a therapeutic strategy for combating RB, with potential prognostic implications.

**Supplementary Information:**

The online version contains supplementary material available at 10.1186/s40364-024-00596-8.

## Background

Retinoblastoma (RB) is the most common primary intraocular malignancy in infancy and children [1], with an annual worldwide incidence ranging from 1 in 15,000 to 1 in 20,000 cases [2]. The management of RB involves various treatment modalities such as enucleation, chemotherapy, laser photocoagulation, cryotherapy, brachytherapy, and external beam radiotherapy [3–5]. While significant progress has been made in treating RB confined to the retina, current approaches for advanced RB have shown limited efficacy. Therefore, there is a critical need to explore novel and efficient therapeutic strategies to improve clinical outcomes.

Emerging insights into tumor metabolism have revealed new therapeutic strategies to exploit altered metabolic dependencies, some of which are currently under evaluation in preclinical models or clinical trials [6]. Metabolic reprogramming in cancer cells often involves significant alterations in cellular energetics, leading to increased rates of glycolysis and lactate production [7]. This metabolic shift results in a pronounced decrease in interstitial pH compared to healthy tissues [8], serving as a critical hallmark of cancer and conferring growth, survival, and aggressiveness advantages to cancer cells [9–11]. Targeting lactate transport in anti-tumor therapy might be crucial to prevent tumor progression, particularly in cases of RB with vitreous seeding or optic nerve invasion where traditional treatment approaches have shown limited efficacy. The avascular nature of the vitreous body and its reliance on glucose and lactic acid contribute to the development of a hypoxic microenvironment. Therefore, further investigation is warranted to unravel the metabolic heterogeneity of RB and elucidate the precise role of lactate transporters in this context.

Lactate transport in cancer is mainly facilitated by monocarboxylate transporters (MCTs), particularly MCT1 (encoded by *SLC16A1*), which play a vital role in transporting lactate and protons across the cell membrane [12–14]. Upregulation of MCTs, particularly MCT1, has been observed in various solid tumors, highlighting their significance in cancer biology [15]. Targeting MCTs has shown promise in reversing aberrant cellular proliferation and aggressive behavior in different malignancies [16–21]. However, the therapeutic value of inhibiting lactate transport in RB remains to be fully understood. In this study, we conducted a comprehensive investigation into the metabolic heterogeneity of human RB at the single-cell level and identified the involvement of MCT1 in the progression of RB.

## Materials and methods

### Analyzing metabolic signatures at single cell resolution

The first step involved the collection of scRNA-seq datasets from 4 RB samples in our previous cohort, which can be accessed via the GEO database with accession ID GSE249995 [22]. Additional scRNA-seq datasets from 7 RB samples were retrieved from the GEO database with accession number GSE168434 [23]. Publicly available scRNA-seq datasets from 5 normal retina samples were obtained from ArrayExpress under the accession number E-MTAB-7316 [24]. The procedures for unsupervised clustering analysis, dimensionality reduction, and cell type assignment strictly adhered to established protocols [22]. Next, a computational pipeline was employed to examine metabolic signature at a single-cell level, following established protocols [25]. Briefly, gene expression quantification utilized log2 (TPM + 1), while cell types with fewer than 50 cells were excluded from downstream analyses. Missing data was addressed using imputation and data normalization techniques to mitigate technical noise within the gene expression profiles. Metabolic gene lists and pathways were sourced from the KEGG database. Imputed expression values were then employed for clustering analysis via the t-SNE method. Quantitative metrics were introduced for identifying cell type-specific metabolic programs, and algorithms were formulated for pathway activity calculation and metabolic heterogeneity measurement. In the pathway activity analysis, mean expression levels for metabolic genes across different cell types were computed, and the relative expression levels of each gene within a specific cell type were determined. Pathway activity scores were then calculated as weighted averages of gene expression levels within respective pathways. To ensure the reliability of the data, outliers were excluded based on their relative expression levels, and the significance of pathway activity was assessed using random permutation tests. For additional reference, the computer codes used in this analysis can be found on the GitHub page of the Locasale Lab: https://github.com/LocasaleLab/Single-Cell-Metabolic-Landscape. PCA analysis was executed on log2-transformed TPM (log2(TPM + 1)) values, excluding imputed missing values. The PCA analysis utilized the prcomp function in R. For each metabolic gene, a PCA score was derived by summing the absolute loading values of the gene in the principal components that collectively accounted for more than 80% of variance. This score assessed gene expression variability across cells. Subsequently, genes' PCA scores were ranked in a descending manner, and GSEA analysis was applied to the ranked gene list for identifying enriched metabolic pathways. The GSEA analysis employed the javaGSEA software, accessible at http://software.broadinstitute.org/gsea/downloads.jsp, with the pre-ranked option and default parameters. Hypoxia signature genes were obtained from the HALLMARK_HYPOXIA gene set within the MSigDB, accessible at http://software.broadinstitute.org/gsea/msigdb/index.jsp.

### Clinical samples

Written informed consents were obtained from the family members of the patients, and the study was approved by the Ethics Review Board of Zhongshan Ophthalmic Center (Guangzhou, China). All procedures were performed in accordance with guidelines in the Declaration of Helsinki. Human RB tissues were obtained from the ocular tumor division of Zhongshan Ophthalmic Center.

### Cell lines and reagents

Two RB cell lines, WERI-Rb1 and Y79, and a nontumor human retinal pigment epithelial cell line ARPE-19 (American Type Culture Collection, Manassas, VA, USA) were used in this study. Y79 and WERI-Rb1 cells, and all derivative cell lines produced in this study, were cultured in RPMI 1640 medium (Corning, USA) supplemented with 10% fetal bovine serum (Gibco, Thermo Fisher Scientific, USA) and 1% penicillin-streptomycin (Invitrogen, USA). ARPE-19 and all derivative cell lines produced in this study were cultured in Dulbecco's Modified Eagle Medium/Nutrient Mixture F-12 (DMEM/F-12) (Gibco, Thermo Fisher Scientific, USA) supplemented with 10% FBS and 1% penicillin-streptomycin. All cells were incubated at 37℃ with 5% CO_2_. Cultures were regularly screened for mycoplasma contamination using Hoechst staining. AZD3965 (S7339, Selleck Chemicals, USA) was dissolved in dimethyl sulfoxide (DMSO) to generate a 1 mM initial concentration, aliquoted, and stored at -20℃. The final concentration of DMSO in the medium did not exceed 0.1%.

### Immunofluorescence assay

The paraffin sections were subjected to powerful antigen retrieval solution (P0088, Beyotime, China) before immunofluorescence staining, following established protocols [26]. Cells were grown on poly-D-lysine (PDL; Sigma) coated coverslips, fixed with 4% paraformaldehyde for 15 minutes, and treated with 0.2% Triton X-100 for 10 minutes at 37℃. Then, the samples were blocked with 5% bovine serum albumin for 30 minutes. Antibodies specific for MCT1 (1:200, ProteinTech, 20139-1-AP), phospho-AMPKα (1:200, Cell Signaling Technology, #50081), phospho-mTOR (1:500, ProteinTech, 67778-1-Ig), phospho-S6 Ribosomal Protein (1:800, Cell Signaling Technology, #5364) and Ki67 (1:2000, ProteinTech, 27309-1-AP) were applied to the samples or sections. Primary antibodies were visualized using species-specific fluorescent secondary antibodies and the nuclei were stained using Mounting Medium with DAPI (ab104139, Abcam, USA).

### siRNA transfection of cell line

Small interfering RNA (siRNA) targeted to MCT1/SLC16A1 (siSLC16A1-1: Sense:5′-GCAGUAUCCUGGUGAAUAATT-3′; Antisense: 5′-UUAUUCACCAGGAUACUGCTT-3′; siSLC16A1-2: Sense:5′-CUGCGAUCCGCGCAUAUAATT-3′; Antisense: 5′-UUAUAUGCGCGGAUCGCAGTT-3′ ; siSLC16A1-3: Sense:5′-GACCAUGAUUGGCAAGUAUTT-3′; Antisense: 5′-AUACUUGCCAAUCAUGGUCTT-3′) and a nonspecific control siRNA (siNC: Sense:5′-UUCUCCGAACGUGUCACGUTT-3′ ; Antisense: 5′-ACGUGACACGUUCGGAGAATT-3′) were designed and validated by HanYi Biosciences lnc (Guangzhou, China). The siRNA transfections were performed using 100 nM siRNA with HiPerFect reagent (301704, Qiagen, Netherland) according to the manufacturer’s transfection protocol of suspension cell lines.

### Cell viability assay

The Cell Counting Kit-8 (CCK-8) assay (A311-01, Vazyme, China) was used to test cell viability. Briefly, cell lines were seeded at a density of 5000 cells per well in 96-well plates, and subsequently treated with varying concentrations of AZD3965 and siRNA. Following the incubation period, 10 µl of the CCK-8 reagent was added to each well and the plates were incubated for an additional 4 hours in the dark. The absorbance of the samples was then measured at a wavelength of 450 nm. All experiments were performed in triplicate to ensure reproducibility of the results.

### EdU proliferation assay

Cell proliferation was assessed using the BeyoClick™ EdU Cell Proliferation Kit with Alexa Fluor 488 (C0071, Beyotime, China). Specifically, cells were seeded onto PDL-coated coverslips in 6-well plates at a density of 1×10^5^ cells/well for 24 hours after transfection or AZD3965 treatment. Following a 2-hour incubation with 10 μM EdU at 37°C, cells were fixed in 4% paraformaldehyde for 15 minutes, treated with 0.1% Triton X-100 for 10 minutes, and rinsed with phosphate buffer saline (PBS) thrice. Subsequently, cells were exposed to 100 μl of click reaction cocktail for 30 minutes, and then stained with Hoechst 33342 for 10 minutes. Fluorescence images were captured using a fluorescence microscope (XD-RFL, SOPTOP, China) with identical exposure settings and analyzed using ImageJ (version 1.46). Edu-positive proliferated cells were quantified by analyzing three randomly selected fields under a fluorescence microscope (400×). The percentage of proliferated cells was determined by calculating the average of Edu-positive nuclei divided by the total number of nuclei in the field.

### Western blotting

The protein from tumor cells and tissues was extracted utilizing RIPA lysis buffer (strong) (K1020, APE×Bio, USA). Subsequently, western blotting was carried out following established procedures [27], using specific antibodies for MCT1 (1:10000, ProteinTech, 20139-1-AP), AMPKα (1:1000, Cell Signaling Technology, #5831), phospho-AMPKα (Thr172) (1:1000, Cell Signaling Technology, #50081), S6 ribosomal protein recombinant antibody (1:5000, ProteinTech, 80208-1-RR), phospho-S6 Ribosomal Protein (Ser240/244) (1:1000, Cell Signaling Technology, #5364), mTOR (1:1000, Cell Signaling Technology, #2983), phospho-mTOR (Ser2481) (1:1000, Cell Signaling Technology, #2974), β-tubulin (1:5000, ProteinFind, HC101-01), and GAPDH (1:10000, ProteinTech, 10494-1-AP). The bound antibodies were subsequently detected by incubating membranes with HRP-conjugated Affinipure Goat Anti-Rabbit IgG(H+L) (1:10000, ProteinTech, SA00001-2) or HRP-conjugated Affinipure Goat Anti-Mouse IgG(H+L) (1:10000, ProteinTech, SA00001-1) for 1 hour, followed by 3 rounds of 10-minute washing in TBS-0.1% Tween-20. Bands were visualized using enhanced chemiluminescence (ECL; Tanon, China) and the Tanon 5200 MultiImage System (Tanon, China).

### Measurement of intracellular lactate level

The intracellular lactate content was quantified using the Lactate Colorimetric Assay Kit (BC2230, Solarbio, China). Following cell lysis with lactate extract buffer, the intracellular lactate levels were measured in accordance with the manufacturer's instructions. Specifically, a total of 5 × 10^6^ cultured cells were washed with PBS and the resulting supernatant (0.8 mL) was collected in a sterile tube post-centrifugation. Standard curves were constructed per the manufacturer's protocol, and the samples were incubated with the reaction mixture for 30 min. After centrifugation to remove insoluble materials, the supernatants were dissolved in ethanol and the absorbance was measured at a wavelength of 570 nm. Calculation of lactate levels was based on the standard curve.

### ADP/ATP ratio assay

The ADP/ATP ratio was assessed using the ADP/ATP Ratio Assay Kit (KA1673, Abnova) in accordance with the manufacturer's protocol. In brief, 1 × 10^4^ treated cells were transferred into white opaque 96-well plates. Following this, 90 μL of ATP reagent was added into each well. After a 1-minute incubation period, luminescence (RLU A) was measured using a luminometer. Ten minutes after the initial measurement, luminescence (RLU B) was recorded again. Immediately after the RLU B measurement, 5 μL of ADP reagent was added to each well. After 1 minute, luminescence (RLU C) was quantified using a luminometer. The ADP/ATP ratio was then calculated using the formula: ADP/ATP ratio = ((RLU C) - (RLU B)) / (RLU A). All experiments were conducted in triplicate.

### Terminal deoxynucleotidyl transferase-dUTP nick end labeling (TUNEL) assay

The paraffin-embedded tissue sections were subjected to deparaffinization followed by permeabilization using a proteinase K solution (20mg/ml; ST533, Beyotime, China) at 37℃ for 30 minutes, prior to the TUNEL assay with the One-Step TUNEL Apoptosis Assay Kit (C1088, Beyotime, China) following the manufacturer's protocol. TUNEL-positive apoptotic cells were quantified by analyzing three random fields containing viable tumor cells from the xenograft sections. The percentage of apoptotic cells per tumor-bearing eye was determined by calculating the average of TUNEL-positive nuclei divided by the total number of nuclei in the field.

### LC‒MS analysis

Liquid chromatography‒mass spectrometry (LC‒MS) (Novogene, China) was utilized for mass spectrometry analysis. Cell samples were ground and mixed with an extraction solution, then centrifuged to collect the supernatant, which was dried using a nitrogen blower. The dried sample was reconstituted in a mixed solvent of acetonitrile and water, followed by sonication and centrifugation in an ice-water bath. Mass spectrometric analysis utilized a Waters UPLC BEH Amide chromatographic column (55°C) with a 5 μL injection volume. Electrospray ionization (ESI) source conditions included an ion source temperature of 600°C and a voltage set at -4500V or 5500V. Multiple reaction monitoring (MRM) was employed for scanning. Data processing included peak alignment, retention time correction, and peak area extraction using SCIEX OS software. Metabolite structures were matched utilizing primary and secondary mass spectra and database retrieval, followed by standardization and statistical analysis of the acquired data.

### Mitochondrial membrane potential assessment

The mitochondrial membrane potential (MMP) measurement kit with JC-1 (M8650, Solarbio, China) was utilized following the manufacturer's instructions. Briefly, after transfection and treatment with AZD3965 for 24 hours, 1 × 10^5^ WERI-Rb1 cells were harvested and suspended in 0.5 mL of cell culture medium. Subsequently, 0.5 mL of JC-1 staining working solution was added to the cells and the sample was incubated at 37°C for 20 minutes. The cells were then centrifuged at 600g for 3 minutes at 4°C, washed twice with JC-1 staining buffer, and resuspended in 1 mL of JC-1 staining buffer for detection. The fluorescence intensity was measured using a multifunctional microplate reader (Synergy H1) with JC-1 monomer detected under λex = 490 nm and λem = 530 nm, and JC-1 aggregates detected under λex = 525 nm and λem = 590 nm. The ratio of fluorescence intensity (green JC monomer / red JC aggregates) indicates the depolarization of mitochondria in cells. Representative images were captured under a fluorescence microscope. All experiments were conducted in triplicate.

### Tumor xenograft model

All animal experiments complied with the ARVO Statement for the Use of Animals in Ophthalmic and Vision Research and the protocols were approved by the Institutional Animal Care and Use Committee of Zhongshan Ophthalmic Center. For xenograft experiments, ten BALB/c female nude mice (18-20 g body weight; 4-6-week-old) were obtained from Zhuhai BesTest Bio-Tech Co (Zhuhai, China) and housed under specific pathogen-free (SPF) conditions with climate control. Orthotopic xenografts of RB were established according to the previously described procedure [26, 28, 29]. Briefly, WERI-Rb1 cells (1 × 10^5^ in 1 µl PBS) were injected into the vitreous of the right eye of mice using a 33g Hamilton needle, while leaving the left eye uninjected as a control. To addressed whether xenograft tumorigenicity could be inhibited by AZD3965, intravitreal AZD3965 (1μl, 1nM-10nM-100nM) and saline was immediately administered following Weri-Rb1 xenograft. Mice were sacrificed 28 days after treatment for enucleation. For assessing tumor regression effect on orthotopic xenograft, mice with successful transplantation were randomly divided into two groups (n = 5 per group) on day 14 after intravitreal transplantation. One group received intravitreal injection of AZD3965 (1 μl) at a final concentration of 100nM, while the other group received saline. After 14 days of treatment, all nude mice were euthanized, and their eyeballs were dissected and fixed in 4% paraformaldehyde for paraffin embedding. The tissue sections were subjected to H&E staining, TUNEL assays, and immunostaining. Retinal thickness was assessed by analyzing paraffin-embedded eye sections stained with Hematoxylin and Eosin. Three randomly selected fields spanning from the central to the peripheral retina were examined under high-magnification microscopy. Four measurements of retinal thickness were taken in each field, and the average thickness was calculated accordingly.

### Histological and immunohistochemistry

Hematoxylin and eosin (H&E) staining and immunohistochemistry (IHC) were performed on 4-μm-thick paraffin sections of human or mouse tissues using standard protocols with optimized conditions. The RB samples were histologically examined using H&E staining following standard procedures. Tissue samples for IHC assay were collected from 47 RB patients who received treatment at the Zhongshan Ophthalmic Center. All RB samples were reviewed by specialized pathologists. The IHC staining of RB samples was scored by two authors (J.T. and Y.W.) under the supervision of an experienced pathologist (P.Z. and S.C.) and ophthalmologist (R.L.). The expression of MCT1 (1:600, ProteinTech, cat. 20139-1-AP) was assessed by semi-quantitative methods using the proportion and intensity of stained tumor cells. The percentage of positively stained cells was counted in five randomly selected fields under a light microscope (400×), and staining intensity was graded as negative (0), weak (1+), moderate (2+), or intense (3+). The percentage of stained cells was classified as≤10% (grade 1), 11–50% (grade 2), or>50% (grade 3). The final IHC score was calculated by multiplying the percentage of the stained cells score and the intensity score. The highest score obtained in this study was 9, and the lowest was 0. MCT1 expression was considered high when the IHC score was≥4 and low when the IHC score was<4.

### Data mining of public datasets

Pan-cancer analysis results of The Cancer Genome Atlas datasets were retrieved from the GEPIA website (http://gepia.cancer-pku.cn) [30].

### Statistical analysis

Data are shown as means ± standard deviation (SD). The R software (R Foundation for Statistical Computing, Vienna, Austria, http://www.r-project.org) and GraphPad Prism Software v 8.0.2 (GraphPad, Inc., La Jolla, CA, USA) was employed for data analysis and presentation. Statistical analysis was performed using an unpaired two tailed Student’s t-test, one-way ANOVA, or Fisher’s exact test. Corresponding significance levels are presented in the figures.

## Results

### Metabolic landscape of retinoblastoma at single cell solution

**Fig. 1:**
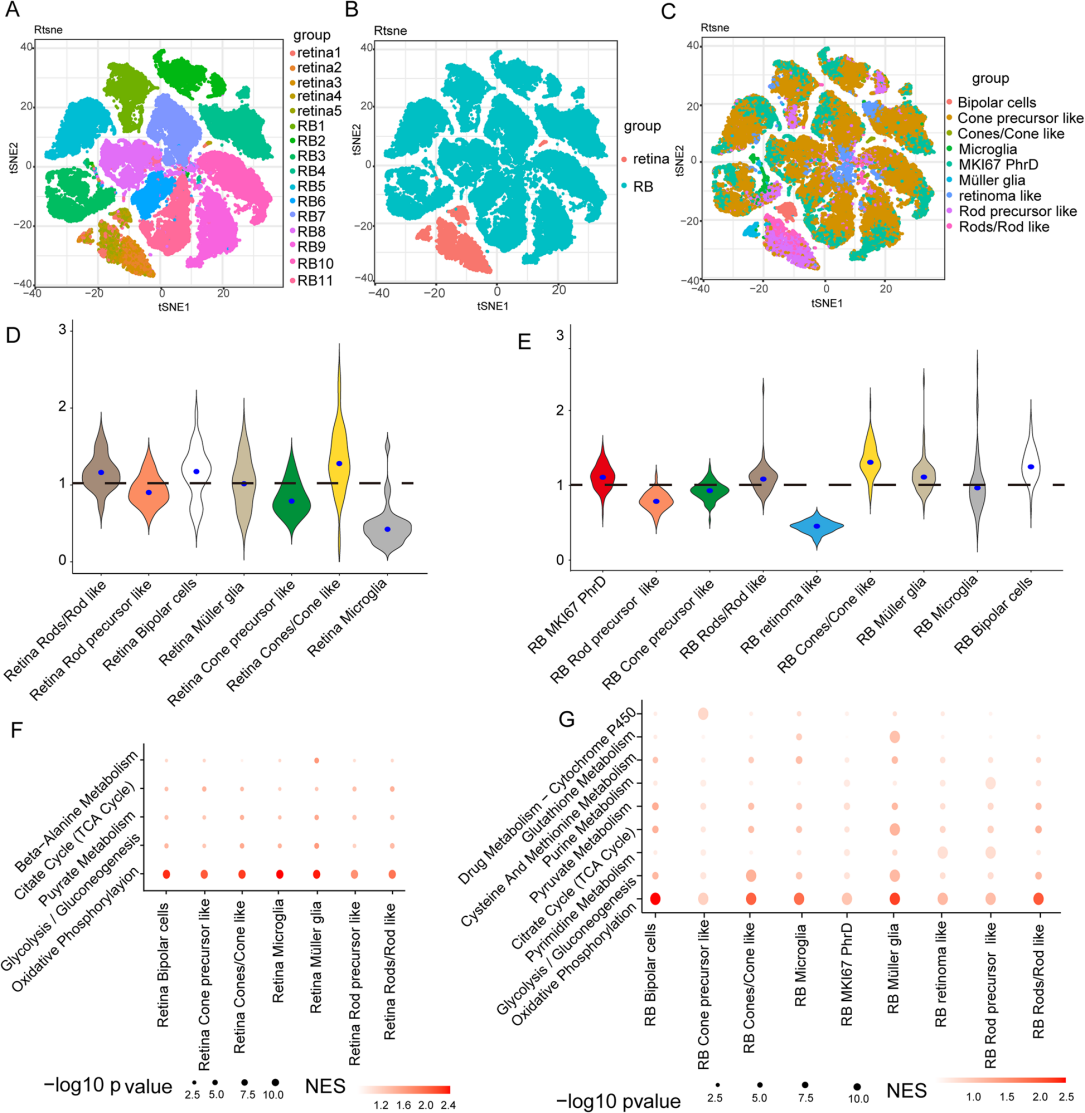
ScRNA-seq analysis reveals distinct metabolic landscape in RB and retina.** A** The t-SNE plot illustrates the metabolic gene expression profiles of both the RB and normal retina datasets. Each dot's color corresponds to the sample origin of the cell. **B** A similar t-SNE plot as in (**A**), but with color-coded representation indicating the tissue type from which each cell originates. **C** Identification of cell clusters in each dataset through analysis of cluster-specific marker gene expression. **D** Distribution of pathway activities across different cell types in the normal retina scRNA-seq dataset. **E** Distribution of pathway activities across different cell types in the RB scRNA-seq dataset. **F** Metabolic pathways enriched in genes with the highest contribution to the metabolic heterogeneities among different cell types in the normal retina dataset. **G** Metabolic pathways enriched in genes with the highest contribution to the metabolic heterogeneities among different cell types in the RB dataset. MKI67 PhrD, MKI67+ photoreceptorness decreased

To characterize the metabolic landscape of RB, we performed a computational pipeline to analyze metabolic signatures at the single-cell level following established procedures [25]. We utilized this comprehensive pipeline to analyze 16 single-cell RNA sequencing (scRNA-seq) datasets derived from 5 human retina samples and 11 human RB samples, which were compiled by integrating our own data with publicly available data [22–24]. The scRNA-seq datasets for human retina encompassed gene expression profiles of 11,080 cells, while the RB datasets contained information from 117,374 cells, making it a comprehensive and extensive resource for studying their metabolic landscape at the single-cell level. Through dimensionality reduction and subsequent clustering analysis using t-distributed stochastic neighbor embedding (t-SNE), we visualized the metabolic gene expression profiles of RB and normal retina. Our results revealed distinct clustering patterns based on metabolic features, indicating a tendency for cells from the same sample to group together, forming cohesive clusters (Fig. 1A). Notably, cells from normal retina samples formed a distinct cluster, while RB samples formed a separate cluster (Fig. 1B). Furthermore, within each sample, different cell types exhibited a tendency to cluster together within their respective clusters (Fig. 1C). Reference marker-based cell-type annotation revealed 9 distinct cell clusters in the scRNA-seq datasets, including MKI67+ photoreceptorness decreased (MKI67^+^ PhrD) cells, cone precursor like cells, rod precursor like cells, cones/cone like cells, rods/rod like cells, müller glia, microglia, bipolar cells, and retinoma like cells. MKI67^+^ PhrD cells represent a highly proliferative population of malignant cells characterized by a significant reduction in photoreceptor differentiation [23].

**Fig. 2:**
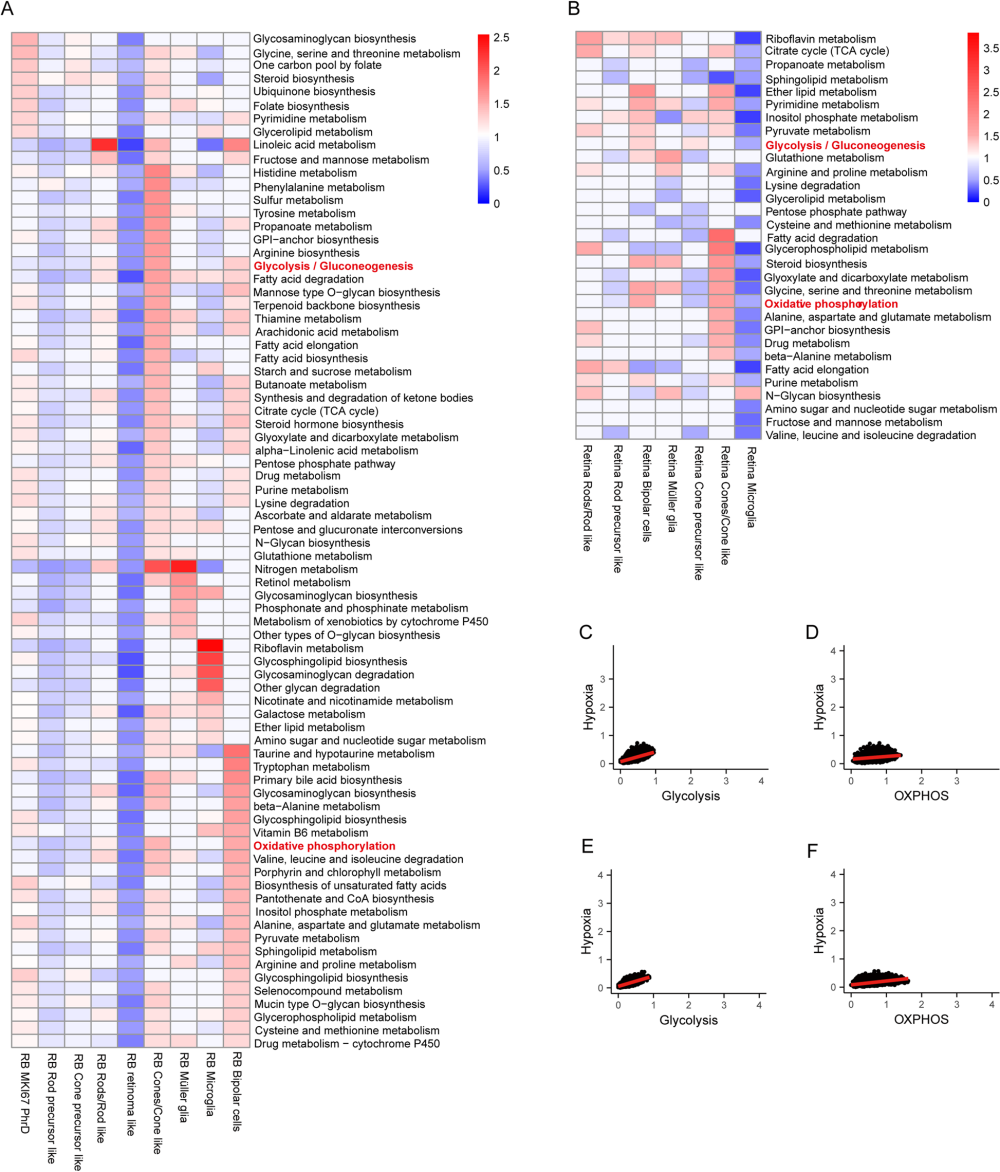
Cell type-specific metabolic reprogramming. **A** Metabolic pathway activities in cell types within the RB datasets. Statistically non-significant values (random permutation test *P* > 0.05) are shown as blank. **B** Metabolic pathway activities in cell types within the normal retina datasets. Statistically non-significant values (random permutation test *P* > 0.05) are shown as blank. **C** Scatter plots comparing activities of glycolysis in response to hypoxia in RB datasets. **D** Scatter plots comparing activities of OXPHOS in response to hypoxia in RB datasets. **E** Scatter plots comparing activities of glycolysis in response to hypoxia in normal retina datasets. **F** Scatter plots comparing activities of OXPHOS in response to hypoxia in normal retina datasets. GPI, Glycosylphosphatidylinositol; MKI67 PhrD, MKI67+ photoreceptorness decreased; OXPHOS, oxidative phosphorylation

We proceeded to investigate the overall variations in metabolic activity among different cell types. In the normal retina scRNA-seq dataset, increased metabolic activity was observed in bipolar cells, rods/rod like cells, and cones/cone like cells (Fig. 1D). Analysis of RB scRNA-seq datasets revealed heightened metabolic activity in MKI67^+^ PhrD cells, bipolar cells, müller glia, rods, and cones, with retinoma cells showing a distinct reduction (Fig. 1E). To identify the key drivers of metabolic heterogeneity, a comprehensive analysis employing principal component analysis (PCA) and gene set enrichment analysis (GSEA) [31] was performed. Both the normal retina and RB prominently showed enrichment of oxidative phosphorylation (OXPHOS) and glycolysis/gluconeogenesis pathways across the majority of cell types (Fig. 1F, G). To assess pathway activity, we computed a pathway activity score by averaging relative gene expression values within each pathway (Fig. 2A, B). Particularly in RB, specific cell clusters, including MKI67+ PhrD cells, rod precursor-like cells, cone precursor-like cells, rods/rod-like cells, cones/cone-like cells, and retinoma-like cells, exhibited upregulated glycolysis/gluconeogenesis compared to OXPHOS (Fig. S1A). In contrast, within the retina, only rod precursor-like cells, cone precursor-like cells, and microglia displayed enhanced glycolysis/gluconeogenesis activity (Fig. S1B). Furthermore, a robust correlation was noted between glycolytic pathway activity and hypoxia across most cell types in both RB and normal retina (Fig. 2C, E). However, this correlation was less pronounced for OXPHOS (Fig. 2D, F). Specific correlation values for the glycolytic pathway, OXPHOS, and hypoxia are detailed in Table S1. These findings emphasize glycolysis's crucial role in tumor metabolic diversity and suggest that lactate, a key glycolytic product, actively influences the tumor microenvironment.

### Overexpression of MCT1 in retinoblastoma

**Fig 3:**
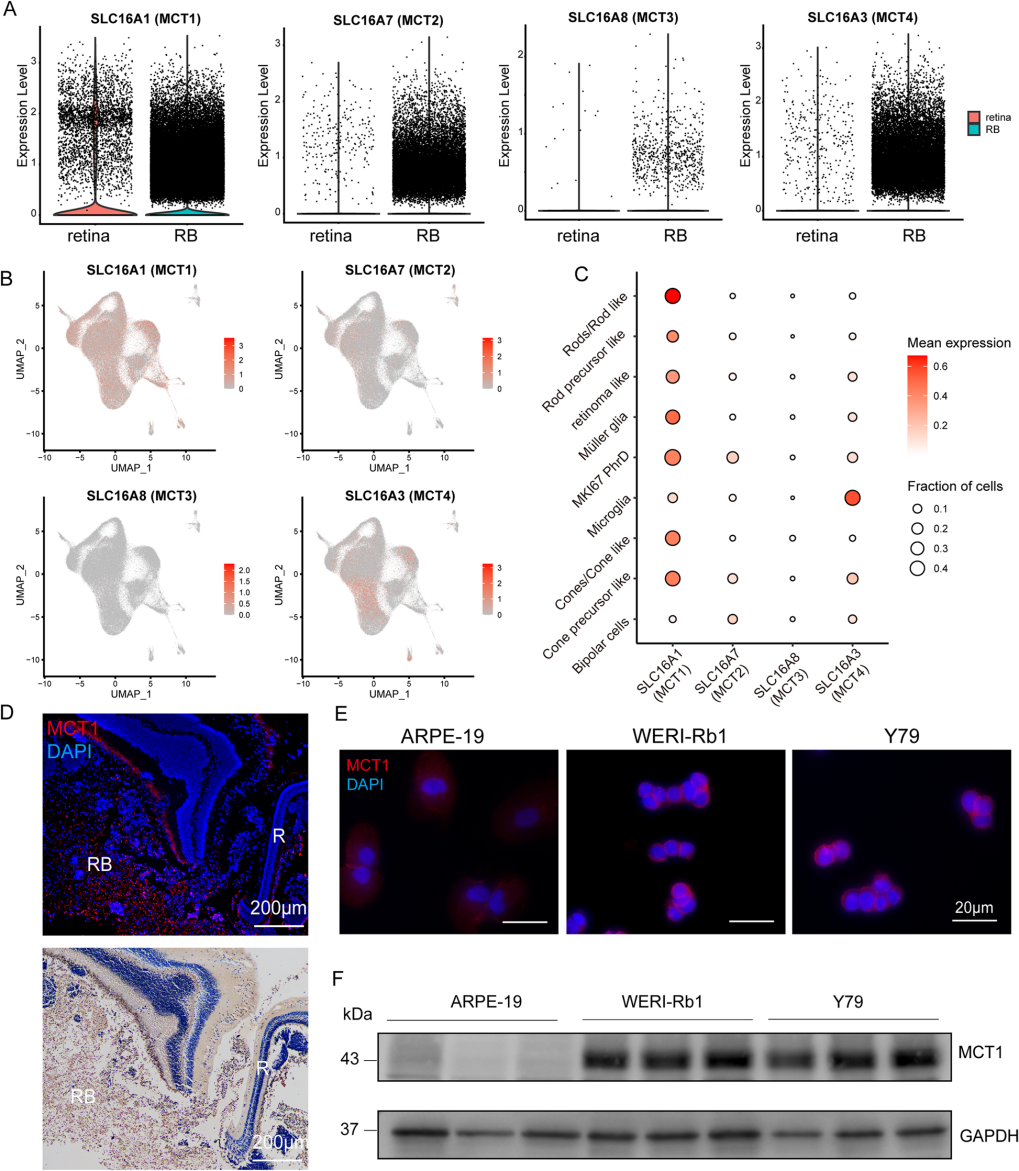
MCT1 is the most expressed monocarboxylate transporter in RB.** A** Comparison of expression levels of different MCTs between RB and retina using scRNA-seq data (SLC16A1 log2FC = 0.226, *P* = 5.17E-191; SLC16A7 log2FC = 0.205, *P* = 1.18E-278; SLC16A8 log2FC = 0.001,* P* = 2.49E-04; SLC16A3 log2FC = 0.265, *P* = 0). **B** Heterogeneous expression patterns of different MCTs across distinct tumor cell clusters, as revealed by scRNA-seq data. **C** Dot plot of normalized expression of different MCTs in the indicated cell types. **D** Immunofluorescence staining and immunohistochemistry staining of MCT1 expression in human RB tissues. **E** Immunofluorescence staining of MCT1 expression in RB cell lines (WERI-Rb1, Y79) and control ARPE-19. **F** Western blot assays and statistical analysis (**G**) of MCT1 expression in RB cell lines and control ARPE-19. The data are reported as the means ± SD (*n* = 3), *P* values were determined by one-way ANOVA, ^****^P < 0.0001. MCTs, monocarboxylate transporters; R, retina; RB, retinoblastoma; MKI67 PhrD, MKI67+ photoreceptorness decreased

To ascertain clinically pertinent candidates implicated in lactate transport, a comprehensive analysis of monocarboxylate transporter expression, encompassing MCT1/2/3/4 (encoded by *SLC16A1/7/8/3*, respectively), was undertaken utilizing scRNA-seq data. Our findings demonstrated a modest upregulation of all four lactate transporters in tumor samples (Fig. 3A). Among these monocarboxylate transporters, MCT1 (*SLC16A1*) and MCT4 (*SLC16A*3) displayed heterogeneous expression patterns across distinct tumor cell clusters (Fig. 3B). Notably, comparing among MCTs, *SLC16A1* exhibited the highest expression levels in MKI67^+^ PhrD cells, rod precursor like cells, cone precursor like cells, rods/rod like cells, cones/cone like cells, retinoma like cells, and müller glia. To further elucidate the functional role of MCT1 in RB, we assessed its expression in human RB tissue samples and RB cell lines (WERI-Rb1, Y79). Immunofluorescence staining and immunohistochemistry confirmed the presence of positive MCT1 expression in human RB tissues (Fig. 3D). Moreover, immunofluorescence staining demonstrated heightened MCT1 expression on the cell membrane of RB cells compared to the control human adult retinal pigment epithelial cell line-19 (ARPE-19) (Fig. 3E). Western blot analysis further confirmed significantly higher MCT1 protein levels in RB cell lines (Fig. 3F).

### MCT1 downregulation alters RB cell viability and energetic metabolism

**Fig. 4:**
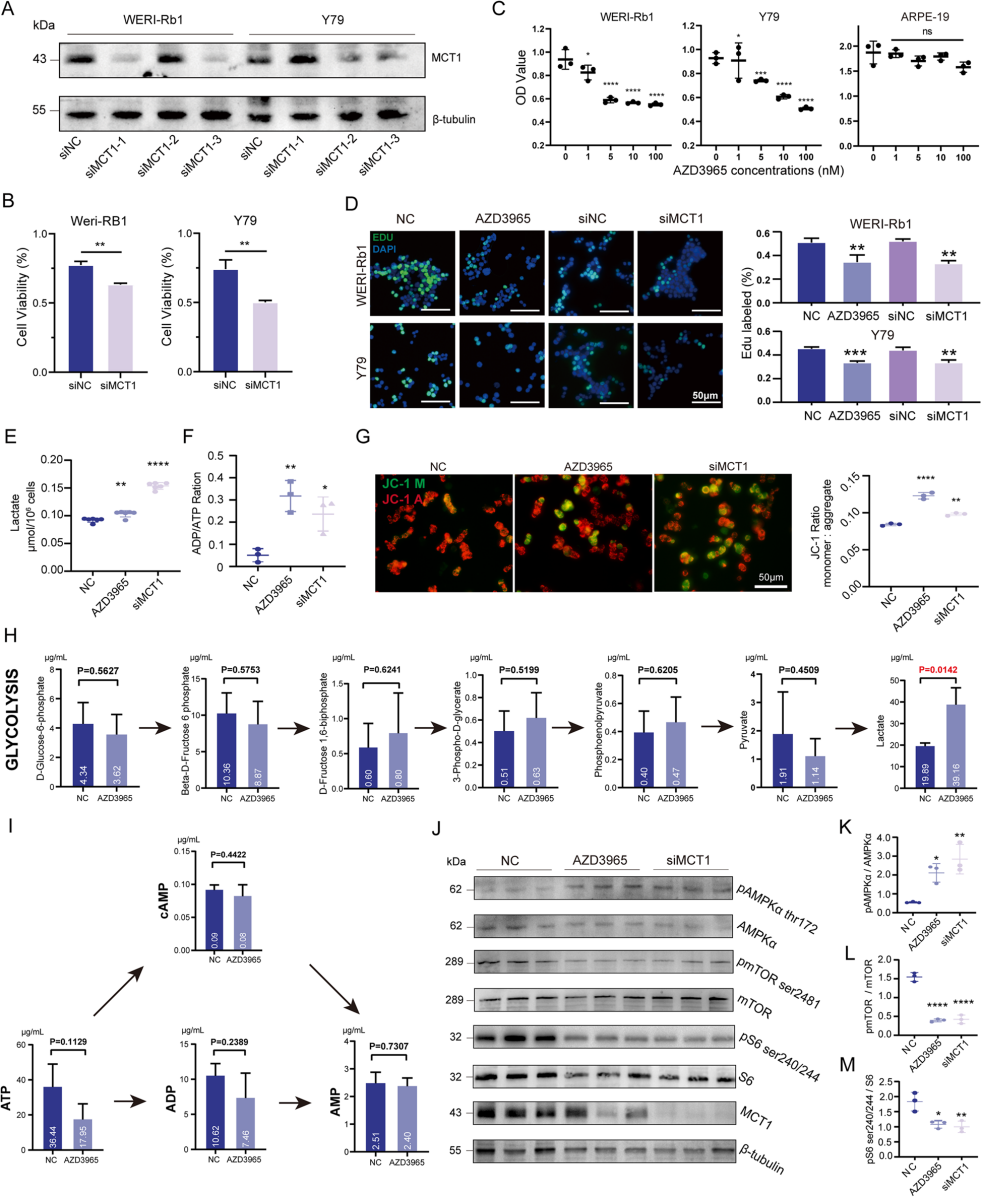
Impact of MCT1 modulation on RB cell viability and metabolic activity. **A** MCT1 knockdown in RB cell lines using siRNA. **B** CCK-8 analysis of RB cell lines after MCT1 knockdown with siRNA for 48 hours. Data presented as means ± SD (*n* = 3), with significance assessed by unpaired, two-tailed Student’s t-test, **P < 0.01. (C) CCK-8 analysis of RB cell lines and ARPE-19 after MCT1 inhibition with AZD3965 in a dose-dependent manner for 48 hours. Data presented as means ± SD (*n* = 3), with significance assessed by ordinary one-way ANOVA, *P < 0.05, ***P < 0.001, ****P < 0.0001. **D** EdU staining analysis of proliferation in RB cell lines after treatment with MCT1 knockdown or inhibition for 48 hours. Data presented as means ± SD (*n* = 3), with significance assessed by ordinary one-way ANOVA, **P < 0.01, ***P < 0.001. **E** Intracellular lactate analysis in WERI-Rb1 cells treated with siMCT1 or 100 nM AZD3965 for 48 hours, compared to negative control. **F** ADP/ATP analysis in WERI-Rb1 treated with siMCT1 or 100 nM AZD3965 for 48 hours, compared to negative control. **G** JC-1 staining in WERI-Rb1 treated with siMCT1 or 100 nM AZD3965 for 48 hours, compared to negative control. Data presented as means ± SD, with significance assessed by ordinary one-way ANOVA. *P < 0.05, **P < 0.01, ***P < 0.001, ****P < 0.0001. **H** Changes in glycolysis metabolites in WERI-Rb1 treated with 100 nM AZD3965 for 48 hours, compared to negative control. **I** Changes in adenosine salvage pathway in WERI-Rb1 treated with 100 nM AZD3965 for 48 hours, compared to negative control. **J** Western blot and semiquantification of expression level changes of (**K**) pAMPK/AMPKα, (**L**) pmTOR/mTOR, (M) pS6/S6, in WERI-Rb1 treated with siMCT1 or 100 nM AZD3965 for 48 hours, compared to negative control

Given the observed MCT1 overexpression in RB, we aimed to explore the functional consequences of inhibiting or silencing MCT1 in RB cell lines. To this end, we utilized AZD3965 to selectively inhibit MCT1 in the cells and designed three independent siRNAs (siMCT1-1, siMCT1-2, and siMCT1-3) to downregulate MCT1. Among these, only siMCT1-3 resulted in a significant decrease in MCT1 expression levels in both RB cell lines (Fig. 4A), and was thus chosen for subsequent experiments. CCK8 assays showed that MCT1 knockdown resulted in a decrease in viable RB cells (Fig. 4B), while AZD3965 treatment caused a dose-dependent reduction in tumor cell viability (Fig. 4C). Notably, treatment with multiple concentrations of AZD3965 for 48 hours had no impact on the viability of ARPE-19 cells, indicating the low cytotoxicity of AZD3965 in vitro (Fig. 4C). Based on the potent anti-cancer effects observed at a concentration of 100 nM, this concentration was used for subsequent experiments. EdU staining further confirmed that MCT1 inhibition or knockdown resulted in a significant decrease in the proliferation of RB cells (Fig. 4D). Considering that MCT1 is primarily responsible for lactate transport, we postulated that the observed decrease in cell viability following MCT1 disruption may be related to aberrant lactate transportation and energy metabolism. Our results revealed that inhibiting or knocking down MCT1 in WERI-Rb1 cells for 48 hours led to intracellular lactate accumulation, alongside an increase in the ADP/ATP ratio and reduced mitochondrial membrane potential (Fig. 4E-G). Mass spectrometry analysis further confirmed that MCT1 inhibition only resulted in a significant increase in intracellular lactate levels within the glycolytic pathway (*P* < 0.05) (Fig. 4H), with a trend towards decreased intracellular ATP content (*P* = 0.1129) (Fig. 4I), while metabolites in the oxidative phosphorylation pathway remained largely unchanged (Fig. S2). AMPK (adenosine monophosphate activated protein kinase) and mTOR (mammalian target of rapamycin) are two crucial signaling pathways that play key roles in cellular energy homeostasis and metabolism. Loss of energy triggers an upregulation of AMPK activity in cells as a mechanism to attenuate the mTOR pathway [32]. Consistent with this, our study demonstrated that MCT1 disruption in WERI-Rb1 cells for 48 hours resulted in increased phosphorylation of AMPKα at Thr172 (pAMPKα), accompanied by decreased phosphorylation levels of mTOR at Ser2841 (pmTOR) and ribosomal protein S6 at Ser240/244 (pS6) (Fig. 4J-M).

### Suppression of retinoblastoma progression in vivo through MCT1 inhibition

**Fig. 5:**
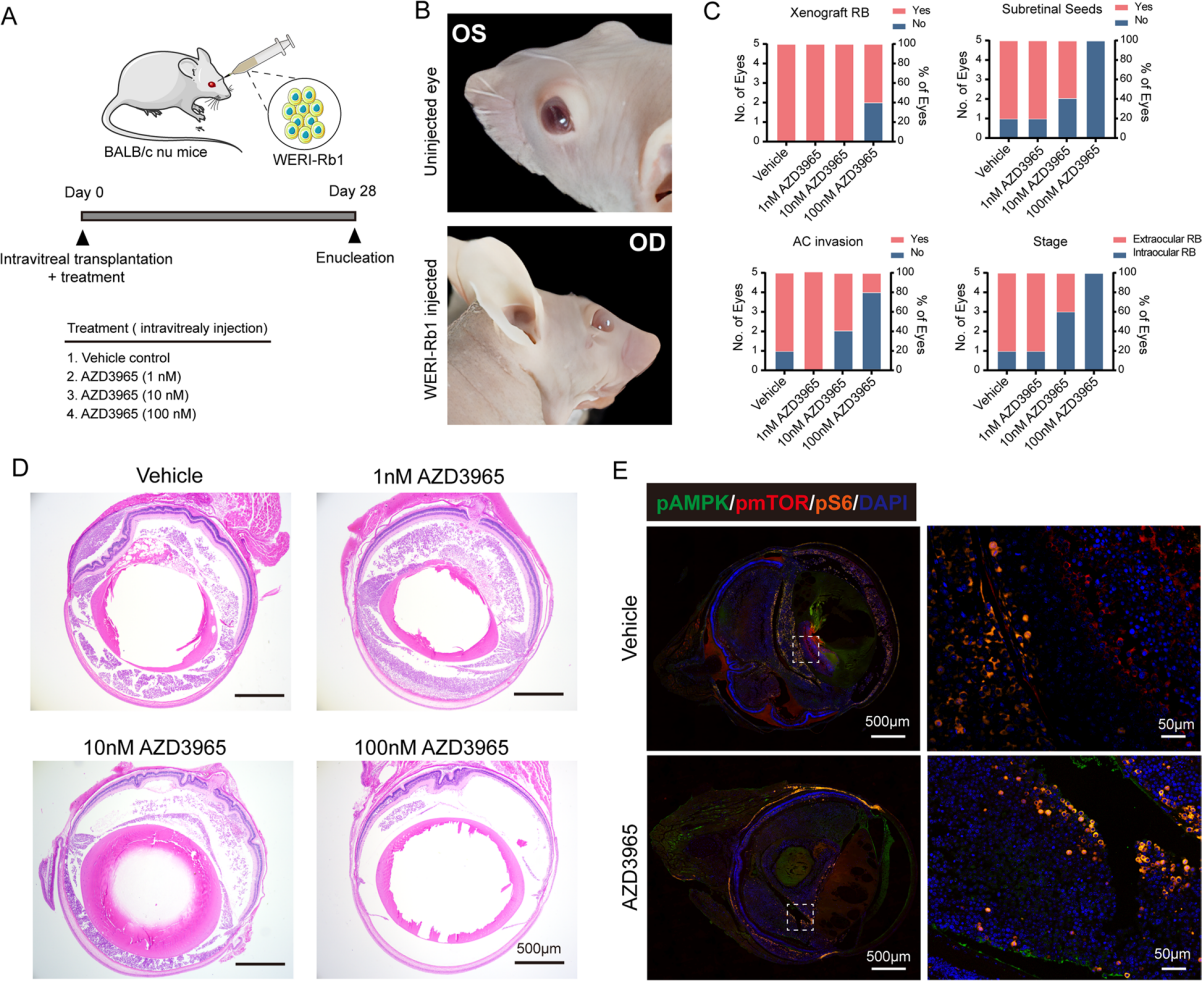
AZD3965 inhibits RB tumor growth in orthotopic xenografts. **A** Experimental design overview. **B** Comparison of WERI-Rb1-xenografted eyes with the uninjected contralateral eye. **C** Incidence of xenograft tumors, subretinal seeds, anterior chamber invasion, and extraocular RB following intravitreal administration of WERI-RB1 cells, coadministered with vehicle (*n* = 5 eyes), 1 nmol AZD3965 (*n* = 5 eyes), 10 nmol AZD3965 (*n* = 5 eyes), and 100 nmol AZD3965 (*n* = 5 eyes) per eye. **D** Representative H&E staining of tumor-bearing eyes from each treatment group. **E** Immunofluorescence staining of pAMPK/pmTOR/pS6 in vehicle and 100 nmol AZD3965-treated groups. OD, right eye; OS, left eye

**Fig. 6:**
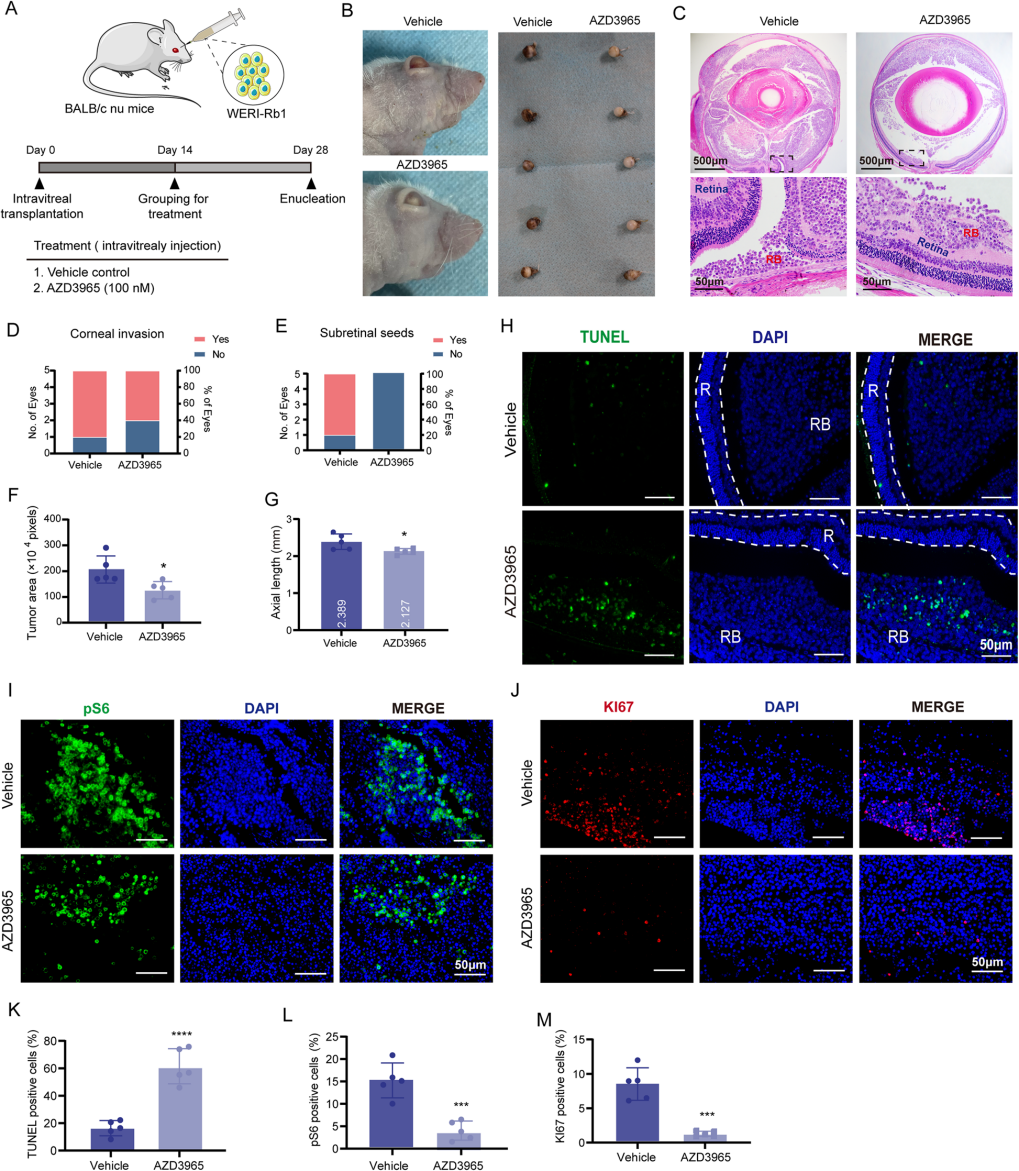
AZD3965 promotes tumor regression in orthotopic RB xenografts. **A** Study schematic. **B** Representative eye views and gross eyeball appearance at 28 days post-xenograft. **C** H&E staining of enucleated eyes from each group. **D**, **E** Proportion of corneal invasion and subretinal seeds in treatment groups. **F** Average tumor areas and (**G**) axial lengths in treatment groups. **H** Representative TUNEL images for each group. RB, retinoblastoma; R, retina. **I** Representative pS6 staining images for each group. **J** Representative Ki67 staining images for each group. **K**-**M** Quantification of apoptotic cells, pS6-positive cells, and Ki67-positive cells in xenografts. Data presented as means ± SD (*n* = 5). Statistical significance determined by unpaired, two-tailed Student’s t-test, *P < 0.05, **P < 0.01, ***P < 0.001, ****P < 0.0001

In order to ascertain the function of MCT1 in RB, we employed a mouse model of cell-derived xenograft by intravitreal injection of WERI-Rb1 cells, followed by immediate administration of varying concentrations of AZD3965 (Fig. 5A). Representative front view images taken on the day of enucleation showed distinct leukocoria in the eyes injected with WERI-Rb1 cells compared to the uninjected eyes (Fig. 5B). Compared to the control group treated with vehicle (PBS) (n = 5 eyes, 100% tumor incidence, 80% extraocular RB, subretinal seeds, and anterior chamber invasion), increasing doses of AZD3965 effectively suppressed xenograft tumorigenesis, with notable effects observed at 100 nM concentration (n = 5 eyes, 60% tumor incidence, 20% subretinal seeds and anterior chamber invasion, and absence of extraocular RB) (Fig. 5C). Additionally, a dose-dependent reduction and altered expression pattern of pAMPK/pmTOR/pS6 were observed in intraocular tumor burden following AZD3965 treatment (Fig. 5D, E). Furthermore, we established an intraocular tumor-regressing model by intravitreal injection of 100 nM AZD3965 (PBS for control group) on day 14 post-initial tumor xenograft (*n* = 5 mice, unilateral cell-derived xenograft) (Fig. 6A). Untreated xenografts displayed prominent tumor features, including protopsis, corneal neovascularization, and leukocoria throughout the 28-day post-transplantation period (Fig. 6B). In contrast, AZD3965-treated eyes maintained retinal layer integrity with lower intravitreal tumor load (Fig. 6C), along with reduced corneal invasion and subretinal seeds (Fig. 6D, E), indicating effective inhibition of tumor progression. Quantitative analysis revealed decreased tumor size and axial length upon AZD3965 treatment (Fig. 6F, G). TUNEL staining showed an increased presence of TUNEL-positive cells within the RB tumor mass of the treatment group (Fig. 6H, K). Importantly, the treatment group did not exhibit a higher level of apoptotic cells in the retina compared to the control group. Retinal thickness analysis showed that both the AZD3965 treatment group and the vehicle group had thinner retinas compared to normal retina. However, only the vehicle group had a statistically significant decrease compared to normal retina, suggesting that AZD3965 treatment did not cause significant retinal damage despite its effectiveness in limiting tumor growth (Fig. S3). The efficacy of tumor regression was further confirmed through pS6 and Ki67 staining, indicating decreased mRNA translation and proliferation within the tumor of AZD3965-treated eyes (Fig. 6I, J, L, M).

### Correlation between MCT1 expression and clinical parameters in retinoblastoma

**Fig. 7:**
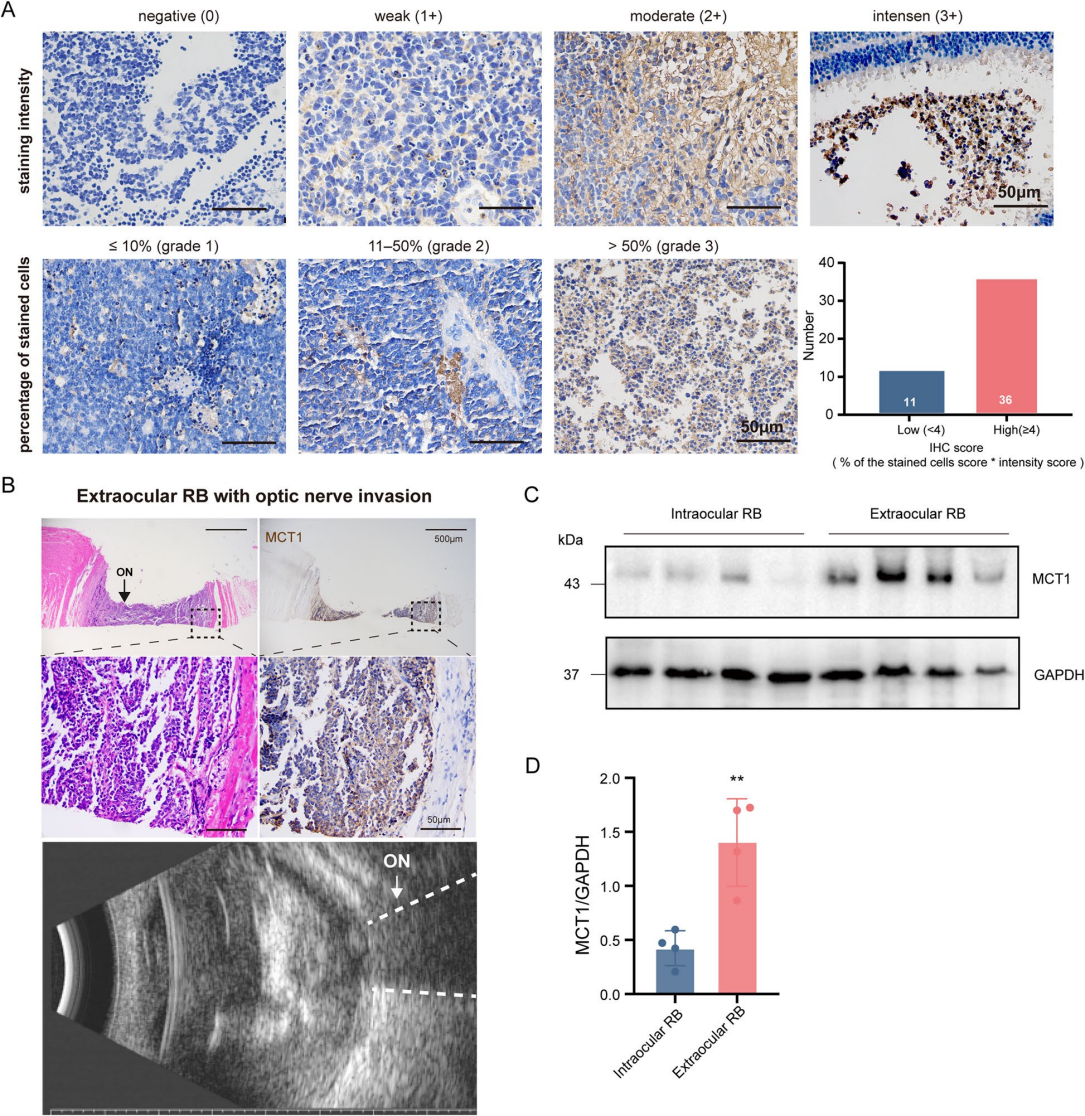
Correlation between MCT1 expression and tumor invasion in retinoblastoma. **A** Representative immunohistochemistry images showing MCT1 protein expression in RB. **B** H&E staining and Immunohistochemistry staining of MCT1 in an extraocular RB patient with post-laminar optic nerve invasion and an enlarged optic nerve in ultrasonography. **C**, **D** Western blot analysis and grayscale analysis of MCT1 expression level changes in intraocular and extraocular RB patients. ON, optic nerve

In order to investigate the expression profile and potential clinical implications of MCT1 (*SLC16A1*) in diverse cancer types, we employed the GEPIA web application. Notably, the analysis revealed a significant upregulation of *SLC16A1* in 12 different malignancies, indicating its potential involvement in tumorigenesis and suggesting a broader role across various cancer types (Fig. S4A). Intriguingly, we also observed a compelling correlation between elevated *SLC16A1* expression and reduced overall survival, indicating its potential as a prognostic indicator in cancer patients (Fig. S4B). To further investigate the relationship between MCT1 expression and clinical parameters of RB development and progression, we conducted MCT1 immunostainings on a cohort of 47 RB samples, comprising 22 males (46.8%) and 25 females (53.2%). Using immunoreactivity scores (IRS), calculated by multiplying positivity and intensity scores, we categorized the samples into two groups: high MCT1 expression (IRS ≥ 4, *n*=36, 76.6%) and low MCT1 expression (IRS < 4, *n*=11, 23.4%) in RB tumors (Fig. 7A). Our findings revealed a significant correlation between MCT1 expression levels and post-laminar optic nerve invasion (*P* = 0.0044), while no specific correlations were observed with other high-risk histopathologic factors (Table 1). Further support for MCT1 expression in the affected optic nerve was obtained through histological staining and immunohistochemical analysis in an extraocular RB patient with post-laminar optic nerve invasion and an enlarged optic nerve in ultrasonography (Fig. 7B). Additionally, we performed western blot analysis to compare MCT1 protein expression in four clinical samples diagnosed with intraocular RB and four samples diagnosed with extraocular RB. The results indicated higher expression of MCT1 protein in extraocular RB compared to intraocular counterparts (Fig. 7C, D).

**Table 1:** Correlation of MCT1 immunoexpression with clinicopathological features in RB

Clinicopathological features	MCT1 immunoexpression (*n* = 47)		
	High *n* = 36	Low *n* = 11	*P*
Sex			
Male (22)	19	3	0.1789
Female (25)	17	8	
Age			
≤ 2 years (22)	16	6	0.7322
> 2 years (25)	20	5	
Massive posterior uveal invasion ( ≥3mm)			
Yes (14)	12	2	0.4643
No (33)	24	9	
Post-laminar optic nerve invasion			
Yes (27)	25	2	0.0044
No (20)	11	9	
Anterior chamber invasion			
Yes (3)	1	2	0.1323
No (44)	35	9	

Staining intensity was recorded from 0 to 3+ (0, no staining; 1+, weak staining; 2+, moderate; 3+, strong) in the cytoplasm of tumor cells. The percentage positivity was scored as 0 (no positive cells), 1 (˂10%), 2 (10–50%) and 3 (˃50%) and each section randomly selected 5 high power fields. The immunoreactivity score (IRS) was then expressed by multiplying the positivity and intensity score. Tumor samples with ≥4 was defined as high

## Discussion

Our study provides a comprehensive analysis of the metabolic landscape in RB at the single-cell level, revealing distinct metabolic features compared to normal retinal cells. Utilizing a computational pipeline to examine scRNA-seq datasets from RB and retina samples, we identified glycolysis/gluconeogenesis as a crucial driver of metabolic heterogeneity within the RB microenvironment. Additionally, our investigation uncovered the overexpression of MCT1 in RB, suggesting its potential role in the metabolic adaptation of RB cells. By inhibiting MCT1, we effectively suppressed RB development and progression, offering a promising strategy for therapeutic intervention. Furthermore, we found a correlation between MCT1 expression and optic nerve invasion in RB samples, hinting at its potential as a prognostic indicator.

Despite previous studies reporting on the presence of a hypoxic tumor microenvironment in advanced RB [33], the characterization of metabolic heterogeneity at the single-cell level within RB remains limited. While the expression levels of metabolic genes may not directly reflect metabolic fluxes or metabolite abundance, emerging evidence suggests that metabolic gene expression holds promise as a predictive indicator of these metabolic processes [34, 35]. Utilizing single-cell gene expression profiles, our study provides insights into the intricate metabolic activities within individual cells. Overall, we observed distinct patterns of metabolic activity between MKI67^+^ PhrD cells and retinoma like cells. MKI67^+^ PhrD cells exhibited heightened metabolic activity, consistent with their characteristic high proliferation rate and genomic instability [36]. Conversely, retinoma like cells showed a notable reduction in metabolic activity, which may be attributed to their low level of genomic instability and elevated expression of senescence-associated proteins [37]. Next, our findings highlight the substantial involvement of OXPHOS and glycolysis in driving the observed metabolic heterogeneity within tumors, enabling tumor cells to adapt and thrive in the challenging tumor microenvironment. The role of OXPHOS and glycolysis activity in shaping tumor metabolism yields several intriguing discoveries. Specifically, the variation in OXPHOS and glycolysis gene expression emerges as a significant contributor to the observed metabolic heterogeneity among cell clusters in both RB and the retina. Furthermore, our metabolic pathway activity analysis revealed a higher glycolysis activity compared to OXPHOS in RB. Another finding from our study is that the activity of glycolysis, rather than OXPHOS, demonstrated a strong correlation with hypoxia in nearly all cell types at the single-cell level. These findings align with the well-established association between glycolysis and hypoxia, which triggers the activation of signal transduction pathways promoting glycolysis while simultaneously suppressing OXPHOS and other mitochondria-associated pathways [38]. Recent investigations have provided evidence of the hypoxic nature of the vitreous gel, wherein oxygen concentration displays a gradient decrease from the retina towards the lens [39]. This hypoxic microenvironment within the vitreous body contributes to the upregulation of glycolytic pathways in RB cells located within the vitreous gel, thereby providing an explanation for the observed higher glycolysis activity relative to OXPHOS in RB.

While the observed elevation in glycolytic activity in RB is noteworthy, it's essential to recognize that normal tissues also depend on glycolysis for regular metabolic functions. As a result, our investigative focus has shifted away from directly targeting the glycolysis pathway. Instead, we are delving into the role of the MCT family in RB. These transporters play a crucial role in facilitating the movement of key glycolytic products, such as lactate, across cell membranes. We have developed a hypothesis centered on the potential hijacking of lactate transporters by cancer cells. This mechanism may serve as a vital component in ensuring an adequate energy supply and facilitating metabolic adaptation within the challenging tumor microenvironment. MCTs are a group of proton-coupled transport proteins responsible for facilitating the transmembrane transport of monocarboxylate metabolites, including pyruvate, L-lactate, and ketone bodies, in conjunction with protons [12]. Among the MCT subtypes, MCT1 and MCT4 play crucial roles in tumor metabolism. MCT1, encoded by the *SLC16A1* gene, mediates bidirectional lactate transport across the cell membrane, driven by concentration gradients [13]. Upregulation of MCT1 has been observed in various human malignancies, including glioblastoma, breast cancer, and neuroblastoma [17, 18, 40]. In contrast, MCT4 facilitates the efflux of lactate produced by glycolysis under hypoxic conditions in tumors and specific cell types like white skeletal muscle fibers and astrocytes [13]. To gain insight into the functional implications of lactate transporters in tumor progression, we performed an analysis of scRNA-seq data. Our analysis unveiled that MCT1 is the most expressed MCTs in MKI67^+^ PhrD cells. Conversely, MCT4 exhibited notable expression in microglia. Furthermore, MCT1 displays lower expression levels in ARPE-19 cells compared to its heightened expression in RB cell lines. However, previous reports have identified MCT1 as the predominant MCT in ARPE-19 cells [41]. The discrepancy between these findings may be attributed to the expression of MCT1 in ARPE19 cells, which may exhibit circadian rhythmicity or be influenced by the growth status of the cells [42, 43]. These findings underscore the potential involvement of MCT1 in RB tumorigenesis and emphasize its significance in facilitating the metabolic adaptations of RB cells. In vitro investigations have demonstrated the involvement of MCT1 in regulating cell growth and proliferation of RB cells. Disruption of MCT1 function hampers the secretion of lactate from cancer cells, resulting in intracellular lactate accumulation and perturbation of the metabolic balance. Notably, this disruption is associated with a decline in ATP levels, reflecting the vital role of MCT1-mediated lactate transport in sustaining cellular energy homeostasis during RB development. AMPK, a cellular energy sensor, is activated in response to various stresses that reduce ATP generation, such as metabolic toxins, nutrient starvation, ischemia, and hypoxia [44]. Additionally, extensive research has demonstrated that mTORC1 activity is modulated by intracellular energy levels through multiple mechanisms, including direct phosphorylation of key components in the mTORC1 pathway by AMPK [45]. Our research revealed that inhibiting MCT1 activity leads to increased activation and phosphorylation of AMPK, while simultaneously decreasing the phosphorylation of mTOR and S6. These findings provide additional evidence of the complex interactions between MCT1 and essential energy-sensing and signaling pathways.

Despite the emergence of novel molecular targeted therapies, the current standard treatment for advanced RB still primarily involves enucleation and chemotherapy [2, 46, 47]. The present study showed the involvement of MCT1 in the tumorigenesis and progression of RB, as the intravitreal injection of MCT1 inhibitors effectively reduces intraocular tumor burden in nude mouse orthotopic transplantation models. Inhibition of MCT1 has shown varying outcomes in different cancer types and has the potential to down-regulate essential glycolytic enzymes and elevate intracellular lactate levels [17, 18, 48]. AZD3965, an MCT1 inhibitor, has recently entered Phase I/II clinical trials (clinicaltrials.gov NCT01791595) for patients with advanced cancers. Despite the ubiquitous expression of MCT1 in various human tissues, the drug exhibited favorable tolerability profiles during the clinical trials, with nausea and fatigue being the most frequently reported adverse effects [49]. Moreover, it is worth highlighting that the anticipated on-target effect on retinal function was observed to be reversible, underscoring the potential for the safe clinical use of AZD3965 [50].

Clinically, high-risk histopathologic factors in RB include tumor invasion into the anterior chamber, an area of massive posterior uveal invasion≥3 mm in diameter, post-laminar optic nerve invasion, or a combination of any non-massive posterior uveal invasion (3 mm in diameter) with any degree of non-retrolaminar optic nerve invasion [51]. During the early stages, optic nerve invasion may only be detectable through microscopic examination [52]. However, its presence becomes critical in predicting the likelihood of extraocular relapse when the tumor extends beyond the lamina cribrosa in enucleated eyes. In our RB cohort, we observed a significant association between elevated MCT1 expression levels and the presence of post-laminar optic nerve invasion. Furthermore, we identified a relatively elevated expression of MCT1 protein in the extraocular RB. In recent years, aqueous humor biopsies have shown notable potential in RB research, covering various aspects such as early diagnosis, tracking tumor response, and monitoring treatment efficacy [53–57]. Recent studies have yielded a wealth of findings regarding AH analysis in RB, uncovering biomarkers such as DNA, RNA, and proteins [58]. Additionally, research has demonstrated that changes in lactate dehydrogenase levels in aqueous humor closely correlate with the clinical and pathological features of RB [59]. Therefore, based on further elucidation of the prognostic role of MCT1 in RB, aqueous humor analysis of MCT1 may be utilized for the early detection of extraocular RB.

This study has several limitations. While we focused on analyzing scRNA-seq data to visualize cellular heterogeneity and metabolic activities within RB, further experimental verification is necessary to validate the functional significance of the identified metabolic pathways and their impact on tumor behavior. Additionally, although our study identified the potential therapeutic implications of targeting MCT1, it is crucial to consider the intricate interplay between metabolic pathways and potential compensatory mechanisms that may limit the efficacy of MCT1 inhibitors as standalone treatments. Moreover, the generalizability of our findings may be restricted to the specific patient cohort included in our study, underscoring the need for validation in larger and more diverse populations. This is further complicated by the challenge of lacking suitable adjacent tissue for comparison and the limited availability of non-retinoblastoma control samples for MCT1 expression analysis. Furthermore, pharmacokinetic studies of intravitreal AZD3965 and the efficacy of systemic AZD3965 were not conducted, and assessments of retinal toxicity via visual evoked potential and electroretinography are lacking. Addressing these limitations in future research is crucial for gaining a more thorough understanding of the therapeutic efficacy and safety profile of MCT1-targeted inhibitors in RB treatment.

## Conclusions

The above findings underscore glycolysis's pivotal role in driving metabolic heterogeneity within RB. Moreover, the observed upregulation of MCT1 in RB, correlating with disease progression, suggests its significance in RB tumorigenesis. Notably, targeting MCT1 emerges as a promising therapeutic avenue for RB.

### Supplementary Information

Supplementary Material 1

## Data Availability

No datasets were generated during the current study.

## Abbreviations

RB: Retinoblastoma
MCT: Monocarboxylate transporter
DMSO: Dissolved in dimethyl sulfoxide
CCK-8: Cell Counting Kit-8
PBS: Phosphate buffer saline
TUNEL: Terminal deoxynucleotidyl transferase-dUTP nick end labeling
LC‒MS: Liquid chromatography‒mass spectrometry
MMP: Mitochondrial membrane potential
SPF: Specific pathogen-free
IHC: immunohistochemistry
scRNA-seq: single-cell RNA sequencing
t-SNE: t-distributed stochastic neighbor embedding
MKI67+ PhrD: MKI67+ photoreceptorness decreased
OXPHOS: Oxidative phosphorylation
AMPK: Aadenosine monophosphate activated protein kinase
mTOR: mammalian target of rapamycin
IRS: Immunoreactivity scores
