# Supplementary material for: Deciphering metabolic heterogeneity in retinoblastoma unravels the role of monocarboxylate transporter 1 in tumor progression
Source: Biomark Res. 2024 May 11;12:48. doi: 10.1186/s40364-024-00596-8 (PMC11088057; doi:10.1186/s40364-024-00596-8)
Supplement: Supplementary file 1 — Supplementary Material 1 [file 40364_2024_596_MOESM1_ESM.docx]

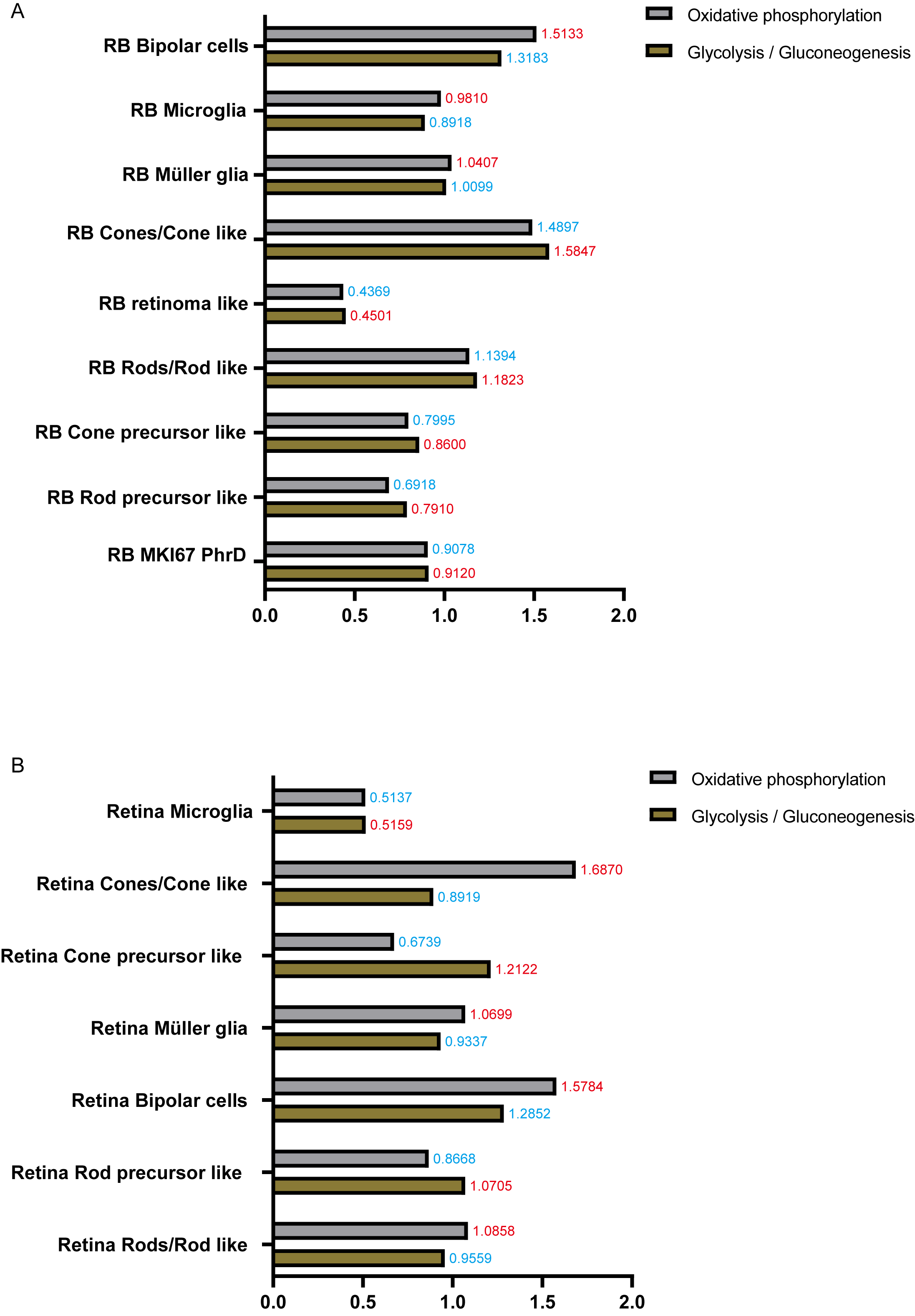


**Fig. S1 Comparison of glycolysis/gluconeogenesis and OXPHOS pathway activities in RB and normal retina** (A) Comparison of glycolysis/gluconeogenesis and OXPHOS pathway activities in RB cell clusters. (B) Comparison of glycolysis/gluconeogenesis and OXPHOS pathway activities in normal retina cell clusters.


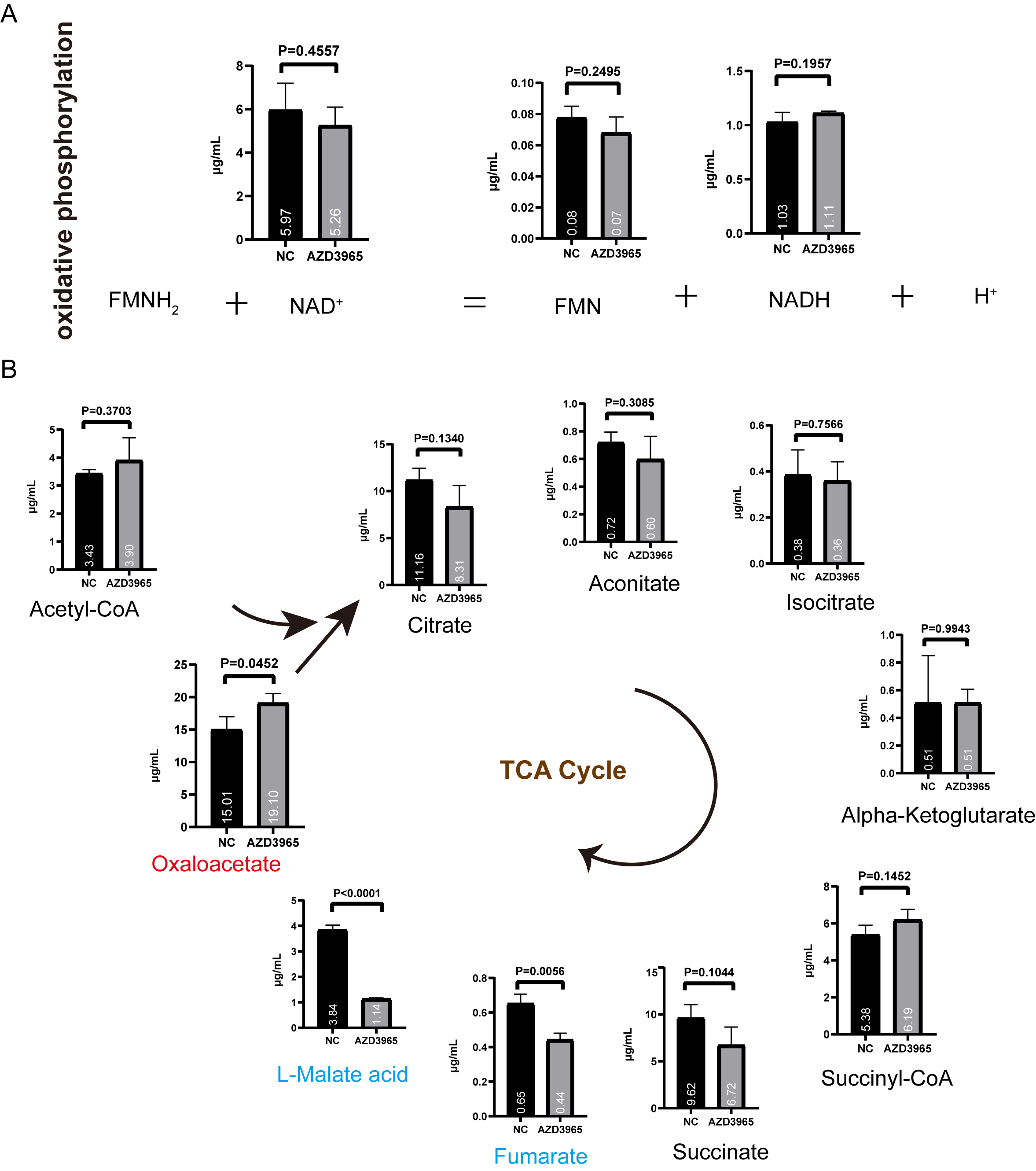


**Fig. S2: Metabolite Levels in Oxidative Phosphorylation and TCA Cycle Analyzed by Mass Spectrometry.** (A) Alterations in oxidative phosphorylation metabolites in WERI-Rb1 cells treated with 100 nM AZD3965 for 48 hours, compared to the negative control. (B) Changes in the TCA cycle in WERI-Rb1 cells treated with 100 nM AZD3965 for 48 hours, compared to the negative control.


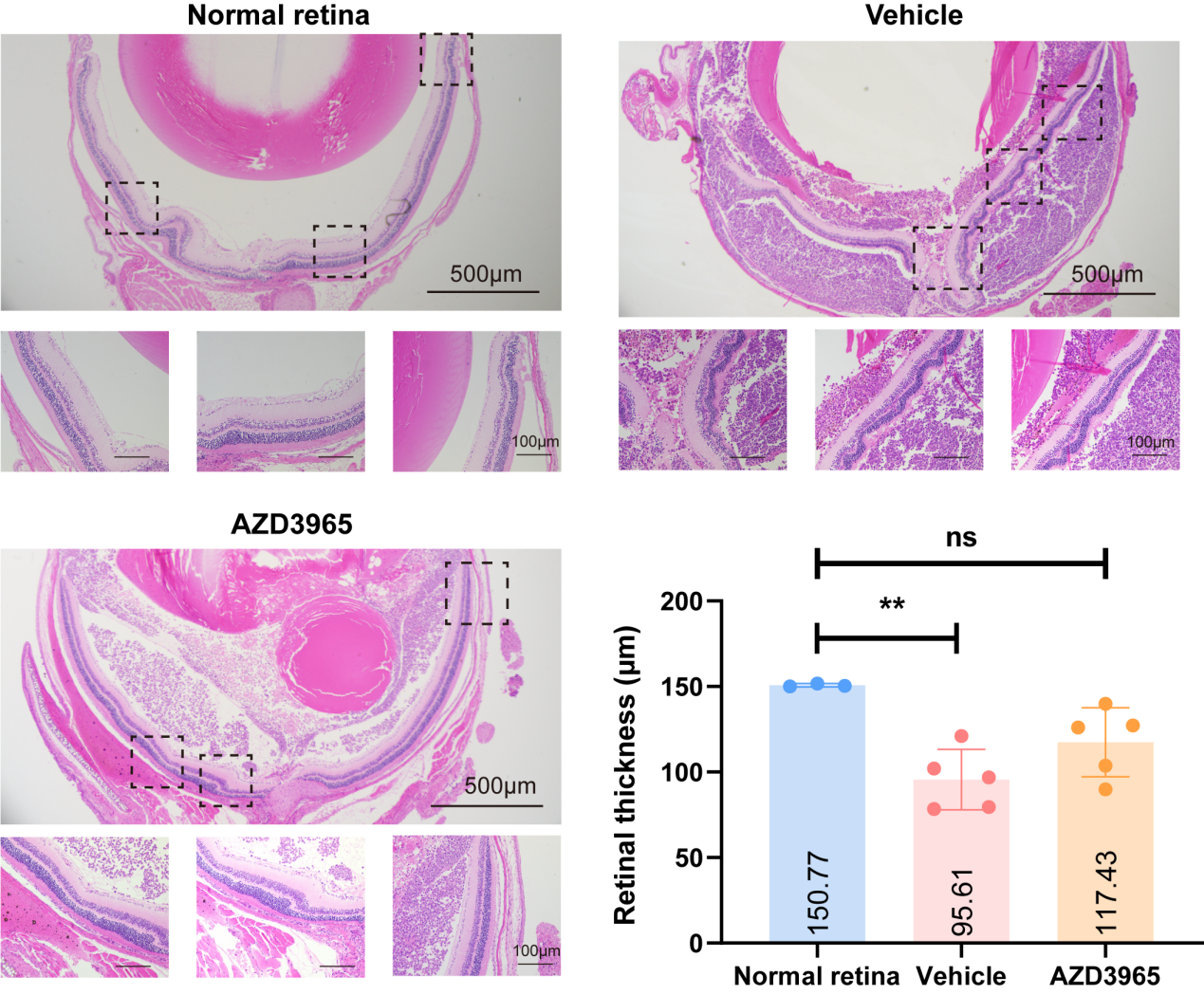


**Fig. S3 Retinal thickness analysis of intraocular tumor-regressing model.** The data are reported as the means ± SD (n = 5 in vehicle and AZD3965; n = 3 in uninjected eye), P values were determined by one-way ANOVA, **P < 0.01.


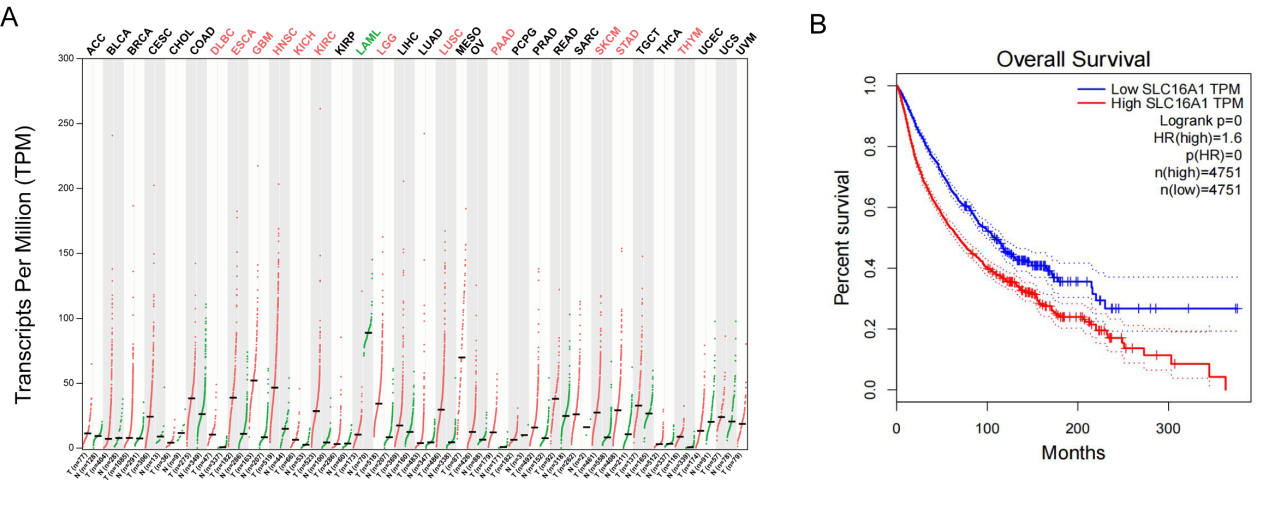


**Fig. S4 Pan-cancer analysis of SLC16A1** (A) Comparison of SLC16A1 mRNA expression among tumor and normal tissues in TCGA. (B) Kaplan-Meier plots of overall survival in patients with low or high SLC16A1 transcripts per million based on the GEPIA database.

**Table S1. Correlation of glycolytic pathway, OXPHOS, and hypoxia in RB and normal retina**

|  | |  | oxphos_hypoxia_cor | glycolysis_hypoxia_cor |
| --- | --- | --- | --- | --- |
| **RB** | MKI67_photoreceptorness_decreased | | 0.06122918 | 0.605190979 |
|  | Rod_precursor_like | | 0.310299357 | 0.709011742 |
|  | Cone_precursor_like | | 0.237255245 | 0.725100464 |
|  | Rods_Rod_like | | 0.480001891 | 0.749670802 |
|  | retinoma_like | | 0.353620304 | 0.684626384 |
|  | Cones_Cone_like | | 0.187655375 | 0.757905228 |
|  | Müller_glia | | 0.405239832 | 0.659898447 |
|  | Microglia | | 0.124968706 | 0.542052121 |
|  | Bipolar_cells | | 0.448567192 | 0.692081347 |
|  |  | |  |  |
| **retina** | Rods_Rod_like | | 0.604945073 | 0.601849546 |
|  | Rod_precursor_like | | 0.341372738 | 0.545818006 |
|  | Bipolar_cells | | 0.457375663 | 0.819317537 |
|  | Müller_glia | | 0.4133042 | 0.683085855 |
|  | Cone_precursor_like | | 0.510734786 | 0.593085026 |
|  | Cones_Cone_like | | 0.413094464 | 0.772022069 |
|  | Microglia | | 0.196705055 | 0.58774455 |
